# Study on the Salivary Microbial Alteration of Men With Head and Neck Cancer and Its Relationship With Symptoms in Southwest China

**DOI:** 10.3389/fcimb.2020.514943

**Published:** 2020-11-06

**Authors:** Hao-Jiang Zuo, Mei R. Fu, Hui-Ling Zhao, Xin-Wen Du, Zi-Yi Hu, Xun-Ying Zhao, Xiao-Qin Ji, Xian-Qiong Feng, Wuerken Zhumajiang, Ting-Hui Zhou, Ya-Li Tian, Xiao-Fang Pei, Rong Yu, Xiu-Ying Hu

**Affiliations:** ^1^West China Hospital/West China School of Nursing, Sichuan University, Chengdu, China; ^2^West China School of Public Health/West China Fourth Hospital, Sichuan University, Chengdu, China; ^3^Boston College William F. Connell School of Nursing, Chestnut Hill, MA, United States; ^4^Medical Statistics and Epidemiology, School of Public Health, Sun Yat-Sen University, Guangzhou, China; ^5^West China Hospital of Stomatology, Sichuan University, Chengdu, China; ^6^Innovation Center of Nursing Research, West China School of Medicine/West China Hospital, Sichuan University, Chengdu, China

**Keywords:** head and neck cancer (HNC), saliva microbiota, 16S rRNA gene sequencing, symptom, geographical biomarkers

## Abstract

This study explored the association between oral microbes and head and neck cancer (HNC) as well as symptoms related to patients with HNC before surgical treatment. Fifty-six patients with HNC and 64 matched healthy controls were recruited from West China hospital in Southwest China. The demographic, clinical, and symptom data were collected. Salivary samples were collected to determine the microbial characteristics using 16S rRNA gene sequencing. Patients with HNC presented increased *Capnocytophaga* abundances. The oral microbial markers as *Capnocytophaga* (area under the curve=0.81) achieved a high classification power between the HNC patients and healthy controls. Moreover, using *Capnocytophaga* in conjunction with symptom of voice/speech difficulty achieved an overall predicting accuracy of 92.5% comparing with using *Capnocytophaga* alone (79.2% accuracy) in distinguishing the HNC patients from healthy controls. Salivary microbial profiles and HNC symptoms may be potential biomarkers for HNC screening.

## Introduction

Head and neck cancers (HNCs) are the common cancers worldwide, accounting for over 650,000 cases and resulting in approximately 330,000 deaths annually (Bray et al., 2018). Currently, surgery or radiotherapy alone or a combination of radiotherapy and chemotherapy are the main treatments for patients with HNC. Unfortunately, due to the lack of effective biomarkers and the complex mechanisms of HNC, approximately 50% of patients with HNC experience local recurrence after primary tumor resection (Liu et al., 2019). Therefore, earlier diagnosis and prognosis for recurrence are urgently needed to increase the 5-year survival rate of these patients.

Studies have shown that the oral microbiota is linked with oral diseases, such as periodontitis, as well as systemic disorders and various cancers (Mager et al., 2005; Yan et al., 2015; Bornigen et al., 2017; Shin et al., 2017; Zhao et al., 2017; Fan et al., 2018a; Hayes et al., 2018; Chang et al., 2019; Mascitti et al., 2019). Based on 16S rRNA gene sequencing on Caucasian participants, Lim et al. found that a panel of *Rothia*, *Haemophilus*, *Corynebacterium*, *Paludibacter*, *Porphyromonas*, *Oribacterium*, and *Capnocytophaga* discriminated patients with oral cavity cancer and oropharyngeal cancers from age-matched controls (Lim et al., 2018). Researchers in northeast China used receiving operational curve (ROC) analysis on the putative throat cancer markers *Pseudomonas*, *Aggregatibacter*, *Bacteroides*, and *Ruminiclostridium6*, and they found robust diagnostic accuracy with an area under the curve (AUC) of 0.875. These results indicated that specific oral microorganisms might be promising noninvasive biomarkers for making an early detection and diagnosis of cancer patients (Zhu et al., 2017; Lim et al., 2018; Wang et al., 2019). However, influence from geographical factors, races and ethnicities, dietary habits, and climates may affect the oral microbial profiles of local people (He et al., 2018). Therefore, data from different geographical areas might be heterogeneous, and the regional backgrounds of oral microbiota should be investigated (Almeida et al., 2019; Mascitti et al., 2019).

Sichuan, one of the most important provinces in Southwest China, has a significantly higher cancer burden than that of other provinces (Zhou et al., 2019). The Sichuan basin has a primarily humid, subtropical climate. Many Sichuan residents have unique eating habits. The food culture is characterized by hemp-and-spicy food, high-fat-and-salt food, and hot food, which may be associated with increased cancer incidence (Chen et al., 2017; Tian et al., 2018). In addition, infection of human papillomavirus (HPV), a risk factor for HNC, was prevalent among HNC patients in Southwest China (Yang et al., 2019). Hence, the oral bacterial profiles of patients with HNC in Southwest China might have some geographical differences compared with profiles in the published studies from other geographic areas in China and other countries (Lim et al., 2018; Wang et al., 2019).

During cancer diagnosis and treatment, patients’ symptoms are essential to ensure the accurate diagnosis and effective treatment (Fu et al., 2016; Hu et al., 2020). In the omic era, microbiome play an important role in symptom science research (Corwin et al., 2019; Fu et al., 2020). Nevertheless, little is known about the relationship between the oral microbiota and symptoms among patients with HNC. In addition, sex-specific differences exist in microbiota compositions (Fransen et al., 2017). HNC is more common in men than in women, with a ratio of 3:1 (Mourad et al., 2017); thus, sex is a critical risk factor of HNC (Mourad et al., 2017), and sex hormones are thought to be one of the imperative factors in altering HNC initiation and progression (Nainani et al., 2014). This study was conducted to explore the oral microbial characteristics and novel biomarkers for male HNC patients in Southwest China, and assess the association between oral microbes and HNC-related symptoms before surgical treatment.

## Materials and Methods

### Participants Population

The study population included men diagnosed with HNC as per National Comprehensive Cancer Network clinical guideline (Lydiatt et al., 2017; NCC, 2019) at West China hospital and healthy men with no cancer diagnosis or other severe illnesses form around communities. Inclusive criteria for patients with HNC were (a) patients with primary tumors in the head and neck area who were scheduled for surgical treatment; (b) patients without intracranial and intraocular tumor; (c) patients who could read and understand Chinese; and (d) patients who could understand and willing to sign the informed consent. Healthy age-matched men were recruited as controls. Participants who met the following criteria were excluded (a) history of prior malignancy and chemotherapy or radiotherapy; (b) history of viral infection (i.e., HBV, HCV, or HIV). The Institutional Review Board of West China Hospital, Sichuan University approved the study (IRB Number: 20171222). All participants signed informed consent.

### Phenotype Data Collection

The demographic and clinical data were collected. Symptom data were collected using the MD Anderson Symptom Inventory-Head and Neck Module-Chinese version (MDASI-H&N-C) (Rosenthal et al., 2007; Han et al., 2010) before surgery.

### Saliva Collection, DNA Extraction, and Illumina MiSeq Sequencing

Before the patients underwent surgery, saliva samples were collected using an adopted saliva sample collection method (Navazesh, 1993; Fan et al., 2018b). Subjects refrained from eating for at least 30 min before collection. Approximately 3 mL of saliva was collected after it accumulated on the mouth floor, followed by expectoration into a specimen tube. Fresh samples were placed in insulating polystyrene foam containers on an ice bath and transported from the hospital to the laboratory. Each sample was divided into 1.5 mL-aliquots and immediately stored at −80°C.

Total DNA was extracted from saliva samples using a Genomic DNA Extraction Kit with magnetic beads (GenMagBio, Jiangsu, China) according to the manufacturer’s protocol. All DNA samples were quality checked, and the concentration was quantified using a Nanodrop 2000 spectrophotometer (Thermo Fisher Scientific, Wilmington, DE, USA) and a Qubit 3.0 Fluorometer (Thermo Fisher Scientific, Wilmington, DE, USA). Bacterial 16S rRNA gene fragments (V3-V4) were amplified from the extracted DNA using primers 341F(5′-CCTACGGGNGGCWGCAG-3′) and 806R(5′-GACTACHVGGGTATCTAATCC-3′) (Michelsen et al., 2013). PCR reactions were performed using the KAPA HiFi HotStart PCR Kit with dNTPs (Kapa Biosystems, Cape Town, South Africa). Purification and normalization were conducted using Agencourt AMPure XP (Beckman Coulter, Brea, CA, USA). The library quality was assessed using the Agilent Bioanalyzer 2100 system (Agilent Technologies, Santa Clara, CA, USA). After being quantified by real-time PCR, the libraries were pooled at equal concentrations. After denaturation, PhiX (Illumina, San Diego, CA, USA) was added as a sequencing control. Amplicons were subjected to paired-end sequencing on the Illumina MiSeq sequencing platform using PE250 chemical at Genetalks Biotechnology, Changsha, China.

### Amplicon Sequence Processing

After demultiplexing, the sequenced reads were merged with FLASH (v1.2.7) (Magoc and Salzberg, 2011) and quality-filtered with fastp (v0.19.6) (Chen et al., 2018). The high-quality sequences were denoised using the DADA2 (Callahan et al., 2016) plugin in the Qiime2 v2020.2 pipeline (Bolyen et al., 2019) with the recommended parameters to obtains single-nucleotide resolution based on error profiles within samples. DADA2 denoised sequences are known as amplicon sequence variants (ASVs). To minimize the effects of sequencing depth on alpha and beta diversity measure, the number of sequences per sample was rarefied to 8870, yielding an average Good’s coverage of 99.94%. ASVs were assigned to taxa using the naive Bayes consensus taxonomy classifier implemented in Qiime2 and the SILVA 16S rRNA database (v138) with a 70% bootstrap cutoff. The 16S rRNA microbiome sequencing data were analyzed using the online platform of the Majorbio Cloud Platform (https://www.i-sanger.com).

### Data Analysis

Alpha diversity was calculated using observed ASVs, Chao1, ACE, Shannon, and Simpson metrics to compare species richness and evenness using Mothur (v1.30.2) (Schloss et al., 2009). Sample size of each group was evaluated by pan bacteria analysis (Xue et al., 2018). To estimate similarity between samples, beta diversity was evaluated by Bray-Curtis dissimilarity and weighted and unweighted UniFrac metrics and illustrated for clustering with principal coordinate analysis (PCoA) plots. Analysis of similarity (ANOSIM) testing was performed to determine group similarities among saliva bacterial community structures (Fierer et al., 2010; Peng et al., 2019). Linear discriminant analysis (LDA) with effect size measurement (LefSe) was used to explore discriminatorily abundant taxonomic characteristics between men with HNC (HNC_M) and health control (HC_M) (Segata et al., 2011). Microbial taxa with LDA scores >2 and a *P*<0.05 were considered significantly different (Wang et al., 2019). Taxa at different levels were also tested by the Wilcoxon rank-sum test. We constructed a random forest model to explore the potential of the oral microbiome to discriminate HNC patients from healthy controls. The model performance was assessed using receiving operational curve (ROC) analysis. Two-fold cross-validation was conducted, in which the ASV database was randomly divided into two parts for random forest model building (training) and validation (testing) (Gilbert et al., 2012). Phylogenetic Investigation of Communities by Reconstruction of Unobserved States 2 (PICRUSt2) was used to infer saliva microbial functions in association with HNC (Douglas et al., 2020). After that, the Kyoto Encyclopedia of Genes and Genomes (KEGG) pathway abundances derived from the predicted KEGG ORTHOLOGY (KO) abundances were performed with MinPath and the “–no_regroup” option (pathway_pipeline.py) (Ye and Doak, 2009; Douglas, 2017; Douglas, 2019; Kim et al., 2019; Kolbe et al., 2019). Significant differences in KEGG categories between the patients and controls were determined using the non-parametric Mann-Whitney U test. We adjusted for potential confounders such as age, smoking, and alcohol consumption. The odds ratio (OR), 95% confidence interval (CI) and *P*-value were estimated using SPSS 25.0 (SPSS Inc., Chicago, IL, United States). *P*-values were corrected for false discovery rate (FDR). Adjusted *P* -value <0.05 was considered significantly different. KEGG results were visualized by ImageGP (http://www.ehbio.com/ImageGP) (Liu et al., 2020). A Spearman correlation heatmap was used to analyze correlations between the salivary microbiota and the features of patients with HNC (**Table S3**).

## Results

### Participant Characteristics

Fifty-nine men with HNC and 67 healthy age- and sex-matched controls were initially enrolled. Upon confirming the HNC diagnosis, we excluded 1 patient who did not undergo surgery, 1 patient who had a polyp, and 1 patient and 3 controls without qualified sequencing results. Finally, 56 patients and 64 controls were used for the analysis. **Table 1** lists their demographic and clinical information. Among the 56 enrolled patients, 47 (83.9%) had laryngeal cancer, and 9 (16.1%) had hypopharyngeal cancer (**Table 1**). **Table S1** lists their symptoms and severity.

**Table 1:** Participant demographics and clinical characteristics (n = 120).

Demographic Characteristics		HNC^1^n(%)	HC^2^n(%)	Statistics	*P*-value
**Age (Years, Mean, SD)**		61.5±8.8	63.3±9.1	Kruskal-Wallis value=1.5311	0.2159
**Sex[n (%)]**	Male [n (%)]	56(100)	64(100)	–	–
	Female [n (%)]	0(0)	0(0)	–	–
**Ethnicity [n (%)]**	Han	56(100)	64(100)	–	–
	Other	0(0)	0(0)	–	–
**Educational Background [n (%)]**	Illiteracy	1(1.8)	4(6.3)	Fisher’s exact test	0.089
	Primary school	16(28.6)	19(29.7)	–	–
	Middle school	22(39.3)	33(51.6)	–	–
	Senior school	11(19.6)	7(10.9)	–	–
	College or bachelor’s degree or above	6(10.7)	1(1.6)	–	–
**Marital Status [n (%)]**	Single	1(1.8)	0(0.0)	Fisher’s exact test	0.2805
	Married	51(91.1)	63(98.4)	–	–
	Divorced/separated	1(1.8)	0(0.0)	–	–
	Widowed	2(3.6)	1(1.6)	–	–
	Other	1(1.8)	0(0)	–	–
**Employment Status [n (%)]**	Unemployed/retired	35(62.5)	45(70.3)	Chi-square value=0.50642	0.4767
	Employed	21(37.5)	19(29.7)	–	–
**Living Situation [n (%)]**	Live alone	4(7.1)	3(4.7)	Fisher’s exact test	0.7039
	Live with family	52(92.9)	61(95.3)	–	–
**Per Capita Monthly Household Income [n (%)]**	Can hardly make ends meet/≤1000	2(3.6)	6(9.4)	Fisher’s exact test	0.2813
	Make ends meet /≤1000	54(96.4)	58(90.6)	–	–
Smoking History [n (%)] **	No	2(3.6)	17(26.6)	Fisher’s exact test	0.0007
	Yes	54(96.4)	47(73.4)	–	–
**Alcohol History [n (%)]**	No	14(25.0)	25(39.1)	Chi-square value=2.0894	0.1483.
	Yes	42(75.0)	39(60.9)	–	–
**Clinical Characteristics [n (%)]**					
**HNC type /Primary lesion site**	Laryngeal cancer /Larynx	47(83.9)	–	–	–
	Hypopharyngeal cancer/Hypopharynx	9(16.1)	–	–	–
	Other	0(0)	–	–	–
**Clinical stage**	I	22	–	–	–
	II	7	–	–	–
	III	7	–	–	–
	IV	20	–	–	–
**Underwent Biopsy [n (%)]**	Yes	56(100)	–	–	–
	No	0(0)	–	–	–
**Other Chronic Illness, Hypertension [n (%)]**	No	39(69.6)	44(68.8)	Fisher’s exact test	1
	Yes	17(30.4)	20(31.3)	–	–
**Other Chronic Illness, Diabetes [n (%)]**	No	54(96.4)	59(92.2)	Fisher’s exact test	0.4467
	Yes	2(3.6)	5(7.8)	–	–

^1^HNC, patients with head and neck cancer (n = 56); ^2^HNC, healthy controls (n = 64). **P-value < 0.01.

### Characteristics of Sequencing

From the 16S rRNA gene sequencing data of the final 120 samples, 2,881,870 qualified reads were filtered out for downstream analysis. Next, 1,052,400 reads were chosen randomly from each sample with an 8870 read cutoff (the minimum number of seqs/sample). Microbial diversity was analyzed after an identical number of 8870 reads were subsampled from each sample by random rarefaction. Finally, 12680 ASVs were obtained and annotated (**Supplementary File 1**).

### Alpha Diversity and Microbiome Composition Analysis

**Figure 1:**
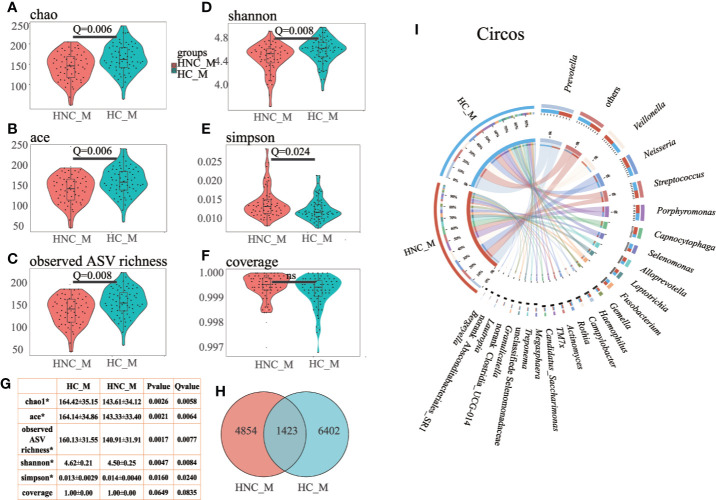
Differential gut microbial characteristics between men with head and neck cancer (HNC_M, n = 56) and healthy controls (HC_M, n = 64). Violin plots of **(A)** Chao1 index, **(B)** ACE index, **(C)** observed ASV richness, **(D)** Shannon index, **(E)** Simpson index, **(F)** Coverage index, **(G)** sum of α-diversity characteristics/Q values, **(H)** Venn diagram, **(I)** Circos plot. The circos plot displaying relative abundances of bacterial orders within the controls and HNC patients. Plots were generated from a rarefied ASV table with all singletons deleted. The abundance of each genus is directly proportional to the size of each band attaching a bacterial taxon and its respective group. Each bacterial genus is assigned a color. The circle shows the accumulative percentage of 16S sequences assigned to a specific genus from each group. (All comparisons, Kruskal–Wallis test.)

Compared with the healthy controls, microbial abundance was significantly decreased in the HNC patients, as estimated by the Chao1 (*P=*0.0026, *Q* = 0.0058) and ACE index (*P=*0.0021, *Q* = 0.0064) with the Wilcoxon rank-sum test. This was also validated by another abundance parameter (observed ASV richness, *P=*0.0017, *Q* = 0.0077). Microbial diversity differed significantly between HNC patients and healthy controls, which was verified by the Shannon (*P*=0.0046, *Q* = 0.0084) and Simpson (*P*=0.0160, *Q* = 0.0240) indices (**Figures 1A–G**). Fewer ASVs were shared among HNC patients than among the controls, suggesting that HNC patients lacked several members of the core microbiome (**Figure 1H**). Further circos analysis of the prevalence and abundance of the specific bacterial genera demonstrated that *Capnocytophaga* (70% vs. 30%) and *Bergeyella* (69% vs. 31%) were more abundant in HNC patients than in the controls. Conversely, *Selenomonas* (38% vs. 62%) was enriched in the controls (**Figure 1I**).

### Beta Diversity Analysis

**Figure 2:**
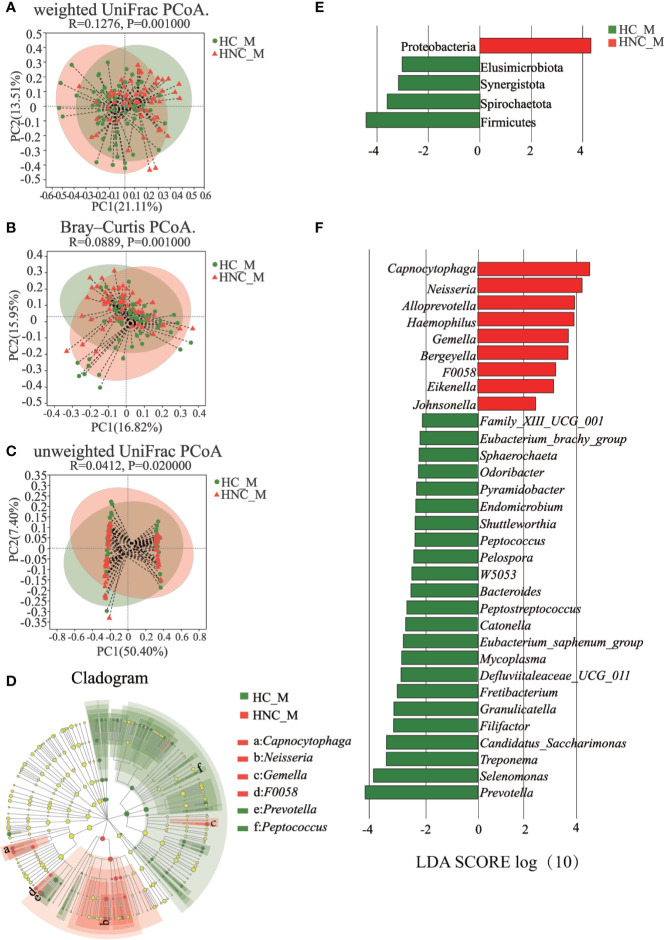
Principal coordinates analysis (PCoA) and LEfSe analysis between men with head and neck cancer (HNC_M, n = 56) and healthy controls (HC_M, n = 64). **(A)** weighted UniFrac PCoA. **(B)** Bray–Curtis PCoA. **(C)** unweighted UniFrac PCoA. *P*-values were determined from 999 permutations in the analysis of similarity test (ANOSIM). **(D)** Cladogram demonstrating the salivary microbiota with significant differences between the two groups. Red and green represent different groups, with the bacterial classifications at the phylum, class, order, family, and genus levels illustrated from inside to outside. The red and green nodes in the phylogenetic tree represent salivary bacteria that play important roles in the HNC patients and healthy controls, respectively. Yellow nodes represent bacteria with no significant difference. **(E)** Linear discriminant analysis (LDA) effect size (LefSe) analysis at the phylum level. **(F)** LefSe analysis at the genus level. LefSe showed a list of specific oral bacteria that enable discrimination between HNC patients and controls. *P*<0.05 and a default LDA score ≥ 2.0 were considered significant in the Kruskal–Wallis and pairwise Wilcoxon evaluation, respectively. The horizontal straight lines in red and green reveal the group means for the HNC patients and controls, respectively. *Capnocytophaga* was the predominant genus in the HNC patients; *Peptococcus* was one of the predominant genera in the controls.

The PCoA results showed that the bacterial communities from the HNC patients and controls were separated from each other based on weighted UniFrac distance (**Figure 2A**, ANOSIM: *P* = 0.001), the Bray–Curtis dissimilarity matrix (**Figure 2B**, ANOSIM: *P* = 0.001), and unweighted UniFrac distance (**Figure 2C**, ANOSIM: *P* = 0.020). LDA and LEfSe were used to select the greatest differences in taxa between HNC patients and healthy controls (**Figures 2D–F**). Based on LDA selection, Proteobacteria (**Figure 2E**) and *Capnocytophaga* (**Figure 2F**) were significantly enriched, while Firmicutes (**Figure 2E**), *Prevotella* and *Peptococcus* (**Figure 2F**) were remarkably reduced in HNC comparing healthy controls. The top 50 bacteria (ASVs) were chosen to differentiate enriched genera within groups. Seven species, including *Prevotella* and *Capnocytophaga*, differed between the HNC patients and healthy controls (**Figure S1A**).

### HNC Prediction

**Figure 3:**
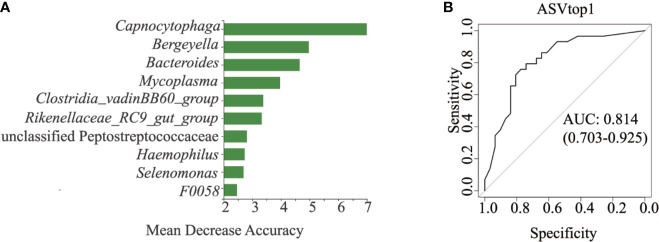
Classification power of specific microbial markers associated with HNC by ROC analysis. **(A)** random forest analysis. Random forest analysis was used to explore the feature contributions (specific microbial markers). **(B)** ROC curves based on the random forest model. The oral microbial markers, such as *Capnocytophaga* (AUC=0.81), achieved a high classification power between head and neck cancer patients (HNC, n = 56) and controls (HC, n = 64). ROC, receiving operational curve; AUC, area under curve; the number of participants in each group.

All 120 samples were randomly divided into two parts, 60 samples for random forest construction, and the other 60 samples for ROC analysis. Abundances of the genus *Capnocytophaga* were able to distinguish HNC patients from healthy controls, with the AUC value 0.81 (95% CI 0.703–0.925; **Figures 3A, B**). When combining the microbiome and symptoms to predict HNC using logistic regression (**Supplementary File 2**), the overall prediction accuracies for *Capnocytophaga* and *Capnocytophaga* + voice symptom were 79.2% and 92.5%, respectively. Compared with the use of the microbiome alone, combining the specific symptoms of HNC with oral microbiome-based classifiers, such as *Capnocytophaga*, was able to better distinguish HNC patients from healthy controls.

### PICRUSt2 Analysis

**Figure 4:**
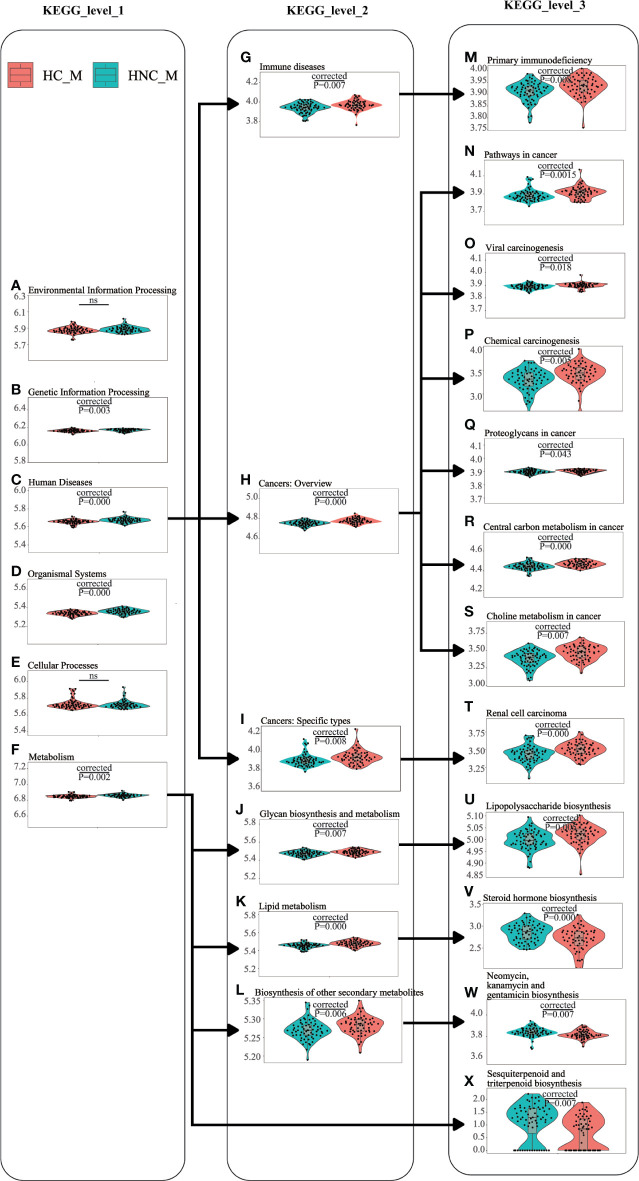
Functional alterations of the salivary microbiome between men with head and neck cancer (HNC_M, n = 56) and healthy controls (HC_M, n = 64) using PICRUSt2. **(A)** Environmental Information Processing, **(B)** Genetic Information Processing, **(C)** Human Diseases, **(D)** Organismal Systems, **(E)** Cellular Processes, **(F)** Metabolism, **(G)** Human Diseases_Immune diseases, **(H)** Human Diseases_Cancers: Overview, **(I)** Human Diseases_Cancers: Specific types, **(J)** Metabolism_Glycan biosynthesis and metabolism, **(K)** Metabolism_Lipid metabolism, **(L)** Metabolism_Biosynthesis of other secondary metabolites, **(M)** Human Diseases_Immune diseases_Primary immunodeficiency, **(N)** Human Diseases_Cancers: Overview_Pathways in cancer, **(O)** Human Diseases_Cancers: Overview_Viral carcinogenesis, **(P)** Human Diseases_Cancers: Overview_Chemical carcinogenesis, **(Q)** Human Diseases_Cancers: Overview_Proteoglycans in cancer, **(R)** Human Diseases_Cancers: Overview_Central carbon metabolism in cancer, **(S)** Human Diseases_Cancers: Overview_Choline metabolism in cancer, **(T)** Human Diseases_Cancers: Specific types_Renal cell carcinoma, **(U)** Metabolism_Glycan biosynthesis and metabolism_Lipopolysaccharide biosynthesis, **(V)** Metabolism_Lipid metabolism_Steroid hormone biosynthesis, **(W)** Metabolism_Biosynthesis of other secondary metabolites_Neomycin, kanamycin and gentamicin biosynthesis, **(X)** Metabolism_Metabolism of terpenoids and polyketides_Sesquiterpenoid and triterpenoid biosynthesis. Violin plots showing the most predominant functional alterations in the HNC group. PICRUSt2: Phylogenetic Investigation of Communities by Reconstruction of Unobserved States 2.

PICRUSt2 with the “–no_regroup” command option (pathway_pipeline.py) was conducted to characterize the functional alterations of the salivary microbiome between HNC patients and healthy controls. Multiple microbial functions were disturbed in the HNC patients, including human diseases at KEGG level1; lipid metabolism at KEGG level2; lipopolysaccharide (LPS) biosynthesis at KEGG level3 (**Figure 4**). Additionally, compared with the healthy controls, rank-sum testing revealed that HNC patients had a significantly higher level of immune system diseases-related functions (*Q*=0.008).

### Associations Between Microbiome and Symptoms

**Table S1** presents the symptoms presence and severity among HNC patients. HNC patients exhibited multiple symptoms preoperatively. The top five most prevalent preoperative symptoms for HNC patients included voice/speech difficulty (80.4%), pain (32.1%), disturbed sleep (26.8%), a feeling of distress (25.0%), and dry mouth (25.0%).

**Figure 5:**
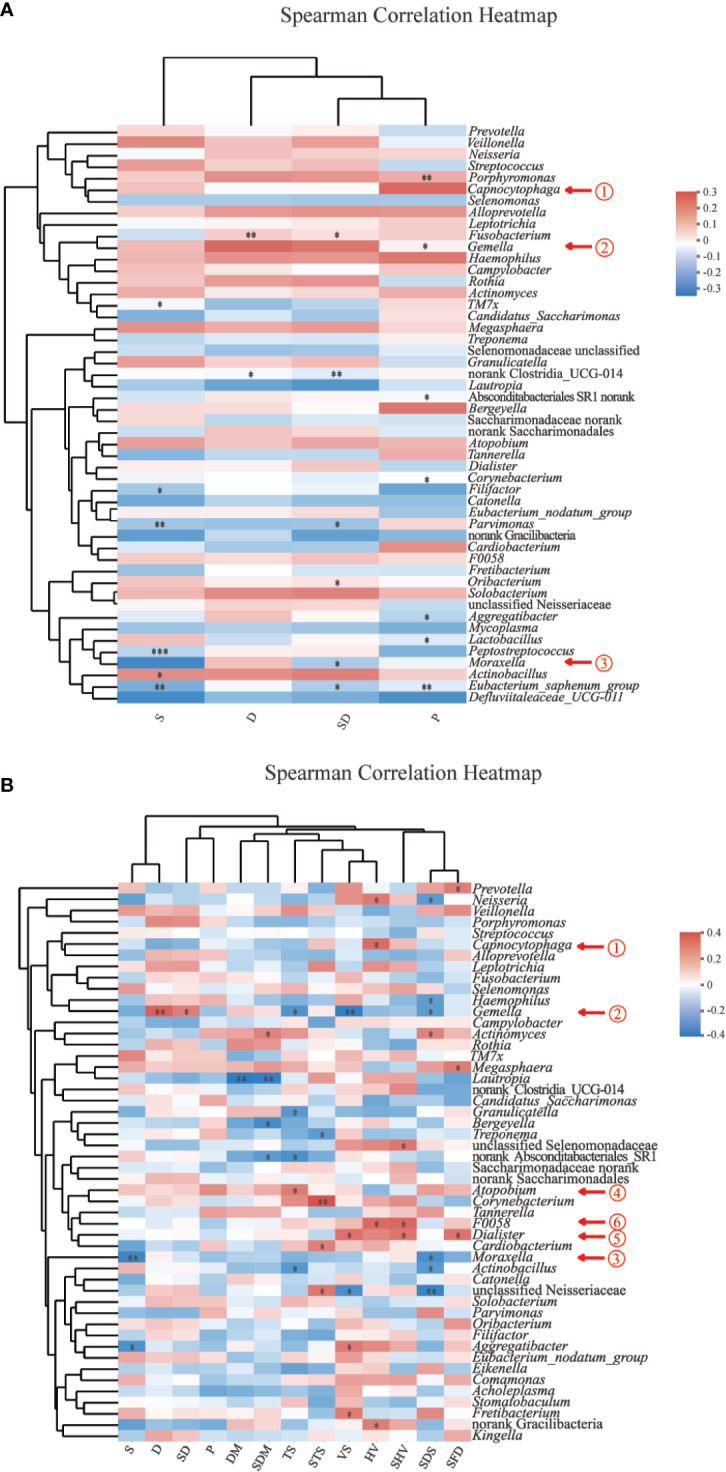
Spearman correlation heatmaps at the genus level. **(A)** Spearman correlation between salivary bacteria and pain symptoms and behavioral habits among all participants (n=120). **(B)** Spearman correlation between salivary bacteria and symptoms and behavioral habits among HNC patients (n=56). Color scheme: red = positive r_s_; blue = negative r_s_; white = r_s_ ∼ 0; r_s_ = Spearman’s rank correlation coefficients, −1< r_s_<1; P, pain; S, smoking; D, drinking; SD, smoking and drinking; VS, voice/speech difficulty; HV, hoarse voice; SHV, the most serious degree of hoarse voice; SFD, The most serious degree of feeling distressed; SDS, The most serious degree of disturbed sleep; TS, thick saliva; STS, the most serious degree of thick saliva; DM, dry mouth; SDM, the most serious degree of dry mouth. *0.01 < *P* ≤ 0.05; **0.001 < *P* ≤ 0.01; ***P ≤ 0.001.

The parameters included in the analysis were voice/speech difficulty, pain, smoking, drinking, and dry mouth. Among all participants (**Figure 5A**), *Capnocytophaga* presence was significantly correlated with pain (*P* =0.006, ρ = +0.25). Among HNC patients (**Figure 5B**), significant correlations were found among all five parameters (voice/speech difficulty, pain, disturbed sleep, being distressed, dry mouth): (1) *Capnocytophaga* and hoarse voice (*P* = 0.012, ρ = +0.33); (2) *Gemella* and drinking (*P* =0.007, ρ = +0.36), drinking and smoking (*P*=0.036, ρ = +0.28), voice/speech difficulty (*P* =0.006, ρ= −0.36), thick saliva (TS, *P*=0.025, ρ= −0.30); (3) *Moraxella* and smoking (*P* =0.009, ρ= −0.35) and the most serious degree of disturbed sleep (*P* =0.021, ρ= −0.31); (4) *Atopobium* and thick saliva (*P* =0.035, ρ = +0.28); (5) *Dialister* and voice/speech difficulty (*P* =0.017, ρ = +0.32), hoarse voice (*P* =0.050, ρ = +0.26), the most serious degree of hoarse voice (*P* =0.023, ρ = +0.30), the most serious degree of feeling distressed (*P*=0.020, ρ = +0.31); and (6) genus *F0058* and hoarse voice (*P* =0.012, ρ = +0.33) and the most serious degree of hoarse voice (*P* =0.010, ρ = +0.33).

Wilcoxon rank-sum testing showed more *Atopobium* in HNC patients with pain than in those without pain (**Figure S1B**, *P* < 0.05, corrected *P* > 0.05). Additionally, more *Neisseria* and *Dialister* were found in HNC patients with voice/speech difficulty than in those without voice/speech difficulty (**Figure S1C**, *P* < 0.05, corrected *P* > 0.05, **Table S2**). Through pan bacteria analysis, the size of subjects in some subgroups (the male HNC patients with voice/speech difficulty, the male HNC patients without pain) are basically sufficient, but some (the male HNC patients without voice/speech difficulty, the male HNC patients with pain) are not sufficient, rarefaction curve indicated that the sequencing depth of each subgroup is sufficient (**Figure S1D–1I**).

## Discussion

The oral microbiota of HNC patients living in Southwest China, which has special regional characteristics, has not been characterized (Chen et al., 2017; Tian et al., 2018). This study explored the oral microbial profiles of HNC patients in Southwest China using 16S rRNA gene sequencing and evaluated the associations between salivary microbial changes and HNC as well as its related symptoms.

Findings of our study showed a significant shift in oral microbiota compositions between HNC patients and healthy controls. Microbial abundance was decreased in HNC patients (**Figure 1**). The oral microbial characterization in HNC patients presented an increase in potential pathogens, including *Capnocytophaga* and other LPS-producing bacteria including *Neisseria*, and a decrease in health-related bacteria, including *Peptococcus* (**Figure 2**) (Si et al., 2017). Importantly, oral microbial markers, such as *Capnocytophaga* (AUC=0.81, **Figure 3B**) achieved a high classification power between HNC patients and healthy controls and may serve as potential noninvasive biomarkers for HNC detection and cancer screen. *Capnocytophaga* consists of various commensal species in the oral flora of humans, including *Capnocytophaga gingivalis* and *Capnocytophaga leadbetteri* (Chen and Chang, 2016). In 2005, Mager *et al*. reported that *Capnocytophaga gingivalis*, *Prevotella melaninogenica*, and *Streptococcus mitis* may be diagnostic markers for predicting 80% of oral squamous cell carcinoma (OSCC) cases (Mager et al., 2005). In 2017, Zhao et al., found that *Capnocytophaga* and 13 other species constituted the oral mucosal core bacteriome of patients with OSCC in Southeast China (Zhao et al., 2017). *Capnocytophaga* was also associated with lung cancer (Yan et al., 2015), lung abscess (Thirumala et al., 2012), brain abscess (Chen and Chang, 2016), and periodontitis, which is causally associated with oral cancer (Bornigen et al., 2017). However, *Pseudomonas*, and not *Capnocytophaga*, was found to be the predominant genus in throat cancer patients in Beijing, China (Wang et al., 2019). *Pseudomonas* was also found to be a unique genus among HNC patients in our study (**Supplementary File 1**). We speculated that regional variation contributed mainly to the microbial variation of HNC patients from different areas of China (He et al., 2018). Here, we confirmed the association between *Capnocytophaga* and HNC patients in Southwest China. Moreover, the combination of *Capnocytophaga* and symptom of voice/speech difficulty enhanced the overall prediction accuracies up to 92.5% (**Supplementary File 2**). Our findings indicated the high accuracy of characterizing oral microbial markers, such as *Capnocytophaga* in conjunction with HNC-related symptoms can be an effective screening tool for HNC, especially for men in Southwest China. Different microbial structures may result in divergent microbial functions and metabolisms, thereby promoting the development and pathogenesis of different illnesses (Mager et al., 2005; Yan et al., 2015; Zhao et al., 2017). PICRUSt2 analysis revealed that the microbial functions involved in human diseases, immune system disease and LPS biosynthesis were increased in HNC patients. These microbial functional alterations at various levels were partly consistent with microbial changes in HNC patients. The increase in immune system disease and LPS biosynthesis may correspond to the increase in *Capnocytophaga* species. Previous study indicated that extracellular enzymes generated by *Capnocytophaga* species could degrade immunoglobulins and complement factors (Alugupalli and Kalfas, 1996). And neutrophil disorder could be induced by *Capnocytophaga* (Shurin et al., 1979). Several studies have also reported that *Capnocytophaga* species can cause illness and severe systemic infections in both immunocompromised and immunocompetent hosts (Parenti and Snydman, 1985; Jolivet-Gougeon et al., 2007; Thirumala et al., 2012). Notably, LPS produced by *Capnocytophaga* strains can produce proinflammatory cytokines, such as IL-1, activate chronic inflammation and oxidative damage, and further lead to cancer development (Kim et al., 1994; Darnaud et al., 2013; Ren et al., 2017). These findings on salivary microbial functions might provide insight into HNC onset and progression.

Emerging evidence shows that different microbiome varied in their associations with clinical symptoms of patients with chronic rhinosinusitis (Wang et al., 2020). Few studies have explored the changes in salivary microbiomes of HNC patients with HNC-related symptoms. Pain and voice/speech difficulty are the most prevalent symptoms of HNC patients (**Table S1**). In our study, we found weak but statistically significant associations between *Dialister* and voice/speech difficulty, hoarse voice, the most serious degree of hoarse voice (**Figure 5B**) were observed. *Dialister* has been associated with endodontic infections, a worse periodontal status and halitosis (Bornigen et al., 2017; Hampelska et al., 2020). Researchers at Johns Hopkins found that patients with oral cancer showed increased populations of *Dialister* relative to those of the controls (Guerrero-Preston et al., 2016). We also found a higher abundance of *Dialister* in HNC patients who had voice/speech difficulty than in patients without voice/speech difficulty (**Figure S1C**). Pain could be directly or indirectly induced by microbial metabolites, neuroactive molecules, cell wall components, and proteins (van Thiel et al., 2020). Symptoms, such as voice/speech difficulty and pain, could hardly be triggered by one or two kinds of oral bacterial microbes. However, precision health applying big data sets that combine omics, such as microbiome, with clinical symptoms could help optimizing disease diagnosis, treatment and prevention specific to different patients (Fu et al., 2020). This pilot study explored the association between saliva microbiome and HNC-related symptoms. Further studies with larger sample sizes and more sophisticated symptom panels could help to find strong associations between microbiome and symptoms. Studies on the components and metabolic consequences deduced from microbial alterations might help better explain the multifactorial etiology of HNC-related symptoms.

Our results cannot be applied to women as this study was conducted only among men to eliminate sex-specific differences in microbiota. Additionally, the comparatively modest sample size may limit the generalizability of the study; however, our sample size was appropriate for the exploratory aims of the study (n_HNC_=56, n_HC_=64). As people living in Southwest China have special dietary habits (Chen et al., 2017; Tian et al., 2018) and high prevalence of HPV (Yang et al., 2019), our results are specific to this geographic population. The cross-sectional design limited the study’s ability to demonstrate a causal relationship between the salivary microbiome and HNC development. Independent validation experiments with clinical HNC samples are needed to further confirm the microbial alterations. For example, the possible causative relationship between the salivary microbiota and HNC development could be further explored *via* conventionalization of germ-free rodents or salivary microbial transplantation to animal models of HNC.

Our study was the first to report salivary microbial characteristics in HNC patients in Southwest China, where people have special dietary habits and a high prevalence of HPV. In our study, HNC patients presented a decreased abundance and diversity in the oral microbiota. The oral microbial characterization in the HNC patients showed an increase in potential pathogens, including *Capnocytophaga* and other LPS-producing bacteria including *Neisseria*, and a decrease in health-related bacteria, including *Peptococcus*. These microbial functional alterations at various levels were partly consistent with microbial changes in HNC patients, possibly owing to the increase in immune system disease and LPS biosynthesis corresponding to the increasing in *Capnocytophaga* species. *Dialister* was closely related to voice/speech difficulty among HNC patients in Southwest China. Moreover, salivary microbes, such as *Capnocytophaga* in conjunction with HNC-related symptoms might be used as a noninvasive tool for HNC screening, detection and treatment monitoring.

## Data Availability Statement

The datasets generated for this study can be found in the NCBI’s Sequence Read Archive under BioProject ID PRJNA 666972, sample accession SAMN16361518-SAMN16361757.

## Ethics Statement

The studies involving human participants were reviewed and approved by the Institutional Review Board of West China Hospital, Sichuan University. The patients/participants provided their written informed consent to participate in this study.

## Author Contributions

MF, H-JZ, X-QF, X-YH, RY, and H-LZ conceived and designed the study. MF, X-YH, RY, X-QF, and Y-LT supported the administrative work of the study. MF, X-YH, and RY contributed to the study material and patient enrollment. H-JZ, X-QJ, X-WD, Z-YH, X-YZ, WZ, T-HZ, and H-LZ contributed to data collection. H-JZ and MF contributed to data analysis. All authors contributed to the article and approved the submitted version.

## Funding

This work was supported by the Senior Visiting Expert Research Funding of West China Hospital (No. 139170042-17248), the Department of Science and Technology of Sichuan Province [2018SZ0404, 2019YJ0018], the Chengdu Science and Technology Bureau [2019-YF05-01247-SN]. The funders had no role in the study design, data collection and analysis, decision to publish, or preparation of the manuscript.

## Conflict of Interest

The authors declare that the research was conducted in the absence of any commercial or financial relationships that could be construed as potential conflicts of interest.

The handling editor declared a shared affiliation with the authors at time of review.

## Supplementary Material

Supplementary File 1 Table of ASVs for 56 HNC patients and 64 healthy controls.

Supplementary File 2 HNC prediction combining microbiome and symptom using logistic regression.

Supplementary Figure 1 The comparative taxonomic profiles, rarefaction curves and pan bacteria analysis among groups. **(A)** Comparative taxonomic profiles of head and neck cancer patients (HNC, n=56) and healthy controls (HC, n=64) at the genus level. Bacteria with significant richness differences (corrected *P* < 0.05) between the two groups are shown. **(B)** Comparative taxonomic profiles of HNC patients with pain (HNCwP, n = 18) and HNC without Pain (HNCwoP, n=38) at the genus level. **(C)** Comparative taxonomic profiles of HNC patients with voice/speech difficulty (HNCwVS, n = 47) and HNC without voice/speech difficulty (HNCwoVS, n=9) at the genus level. **(D)** Rarefaction curves of HNC patients and controls. **(E)** Pan bacteria analysis HNC patients and controls. **(F)** Rarefaction curves of HNC patients and controls. **(G)** Pan bacteria analysis of HNCwP and HNCwoP. **(H)** Rarefaction curves of HNCwVS and HNCwoVS. **(I)** Pan bacteria analysis of HNCwVS and HNCwoVS.
