# Supplementary material for: Study on the Salivary Microbial Alteration of Men With Head and Neck Cancer and Its Relationship With Symptoms in Southwest China
Source: Front Cell Infect Microbiol. 2020 Nov 6;10:514943. doi: 10.3389/fcimb.2020.514943 (PMC7685052; doi:10.3389/fcimb.2020.514943)
Supplement: Supplementary File 1 — Table of ASVs for 56 HNC patients and 64 healthy controls. [file DataSheet_1.zip › Supplementary Table 1.docx]

**TABLE S1.** Symptoms presence and severity of HNC patients

|  | Symptom presence | | Symptoms Severity | |
| --- | --- | --- | --- | --- |
|  | n(%) | rank | Range | M (P25, P75) |
| Pain | 18(32.1) | 2 | 0–5 | 0(0, 2.25) |
| Fatigue | 9(16.1) | 7 | 0–4 | 0(0, 0) |
| Nausea | 0(0.0) | 19 | 0–0 | 0(0,0) |
| Disturbed sleep | 15(26.8) | 3 | 0–6 | 0(0,2) |
| Feeling of being distressed | 14(25.0) | 4 | 0–6 | 0(0,0.5) |
| Shortness of breath | 2(3.6) | 17 | 0–5 | 0(0,0) |
| Problem with Remembering things | 3(5.4) | 14 | 0–3 | 0(0,0) |
| Lack of appetite | 7(12.5) | 8 | 0–5 | 0(0,0) |
| Feeling drowsy | 3(5.4) | 15 | 0–4 | 0(0,0) |
| Dry mouth | 14(25.0) | 5 | 0–8 | 0(0,0.5) |
| Feeling sad | 5(8.9) | 11 | 0–5 | 0(0,0) |
| Vomiting | 0(0.0) | 19 | 0–0 | 0(0,0) |
| Numbness or tingling | 7(12.5) | 8 | 0–4 | 0(0,0) |
| Problem with mucus | 13(23.2) | 6 | 0–7 | 0(0,0) |
| Difficulty swallowing/chewing | 7(12.5) | 8 | 0–5 | 0(0,0) |
| Choking/coughing | 4(7.1) | 13 | 0–5 | 0(0,0) |
| Voice/speech difficulty | 45(80.4) | 1 | 0–9 | 4(2,5) |
| Skin pain/burning/rash | 0(0.0) | 19 | 0–0 | 0(0,0) |
| Constipation | 5(8.9) | 12 | 0–4 | 0(0,0) |
| Problem with tasting food | 0(0.0) | 19 | 0–0 | 0(0,0) |
| Mouth/throat sores | 2(3.6) | 18 | 0–3 | 0(0,0) |
| Problem with teeth or gums | 3(5.4) | 16 | 0–4 | 0(0,0) |
| TSSSs^a^ | - | - | 0–37 | 9(6,16) |

**a.** TSSSs: Total symptom severity score.
