# Supplementary material for: Study on the Salivary Microbial Alteration of Men With Head and Neck Cancer and Its Relationship With Symptoms in Southwest China
Source: Front Cell Infect Microbiol. 2020 Nov 6;10:514943. doi: 10.3389/fcimb.2020.514943 (PMC7685052; doi:10.3389/fcimb.2020.514943)
Supplement: Supplementary File 1 — Table of ASVs for 56 HNC patients and 64 healthy controls. [file DataSheet_1.zip › Supplementary Table 2.docx]

**TABLE S2.** Oral microbiome analysis adjusted for potential confounders

| Taxon | Groups | | OR (95% CI) ^a^ | *P* ^a^ | *Q* ^b^ |
| --- | --- | --- | --- | --- | --- |
| Genus *Capnocytophaga* | HNC_M(n=56) | HC_M(n=64) | 1.004 (1.002-1.006) | 0.000 | 0.000 |
| Genus *Atopobium* | HNCwP(n=18) | HNCwoP(n=38) | 1.001 (0.995-1.007) | 0.015 | 0.038 |
| Genus *Neisseria* | HNCwVS(n=47) | HNCwoVS(n=9) | 1.002(1.000-1.005) | 0.070 | 0.117 |
| unclassified Selenomonadaceae | HNCwVS(n=47) | HNCwoVS(n=9) | 1.022(0.994-1.052) | 0.128 | 0.160 |
| Genus *Dialister* | HNCwVS(n=47) | HNCwoVS(n=9) | 1.012(0.965-1.062) | 0.616 | 0.616 |
| Species *Capnocytophaga_leadbetteri* | HNCwVS(n=47) | HNCwoVS(n=9) | 1.017(1.007-1.028) | 0.000 | 0.000 |

a Odds ratios (OR)s, 95% CIs and *P*-values were calculated via logistic regression. Potential confounders such as age, smoking, alcohol consumption were adjusted.

b False discovery rate corrected *P*-values.
