# Supplementary material for: Study on the Salivary Microbial Alteration of Men With Head and Neck Cancer and Its Relationship With Symptoms in Southwest China
Source: Front Cell Infect Microbiol. 2020 Nov 6;10:514943. doi: 10.3389/fcimb.2020.514943 (PMC7685052; doi:10.3389/fcimb.2020.514943)
Supplement: Supplementary File 1 — Table of ASVs for 56 HNC patients and 64 healthy controls. [file DataSheet_1.zip › Supplementary Table 3.docx]

**TABLE S3. List of analytic methods and software used**

| No. | Analytic method | Software/database |
| --- | --- | --- |
| 1 | denoising | DADA2 plugin |
| 2 | abundance tables generation | Qiime2 (v2020.2) |
| 3 | taxonomy annotation | naive Bayes classifier |
| 4 | 16 rRNA database | SILVA138 |
| 5 | Pan/Core analysis | R-3.3.1, Vegan (v2.5-3) |
| 6 | Rank-Abundance analysis | Python (v2.7) |
| 7 | alpha diversity analysis | Mothur (v1.30.2) |
| 8 | Inter group difference test | R-3.3.1 (stat) |
| 9 | PCoA(principal co-ordinates analysis) | R-3.3.1, Vegan (v2.5-3) |
| 10 | Circos | Python (v2.7) |
| 11 | LEfSe (Linear discriminant analysis Effect Size) | R-3.3.1 |
| 12 | function prediction | PICRUSt2 (Phylogenetic Investigation of Communities by Reconstruction of Unobserved States, v2.2.0-b) |
| 13 | Spearman correlation heatmap | R-3.3.1, Python (v2.7) |
| 14 | Random Forest analysis | R-3.3.1 (randomForest package) |
| 15 | ROC (receiver operating characteristic curve) | R-3.3.1 (pROC package) |
| 16 | logistic regression | SPSS 25.0 |
