# Supplementary figures and images for: Study on the Salivary Microbial Alteration of Men With Head and Neck Cancer and Its Relationship With Symptoms in Southwest China
Source: Front Cell Infect Microbiol. 2020 Nov 6;10:514943. doi: 10.3389/fcimb.2020.514943 (PMC7685052; doi:10.3389/fcimb.2020.514943)

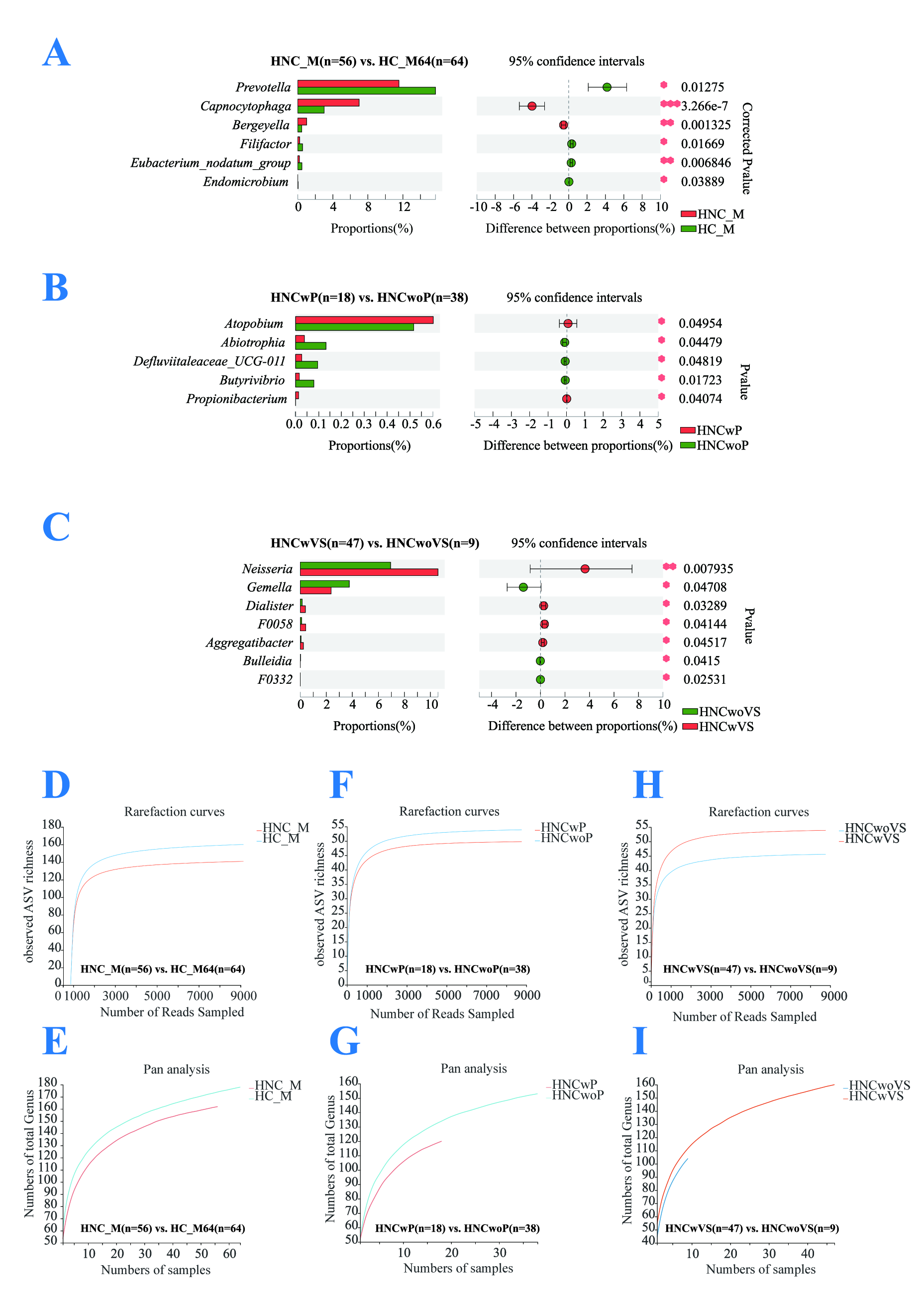

Supplement: Supplementary Figure 1 — The comparative taxonomic profiles, rarefaction curves and pan bacteria analysis among groups. (A) Comparative taxonomic profiles of head and neck cancer patients (HNC, n=56) and healthy controls (HC, n=64) at the genus level. Bacteria with significant richness differences (corrected P < 0.05) between the two groups are shown. (B) Comparative taxonomic profiles of HNC patients with pain (HNCwP, n = 18) and HNC without Pain (HNCwoP, n=38) at the genus level. (C) Comparative taxonomic profiles of HNC patients with voice/speech difficulty (HNCwVS, n = 47) and HNC without voice/speech difficulty (HNCwoVS, n=9) at the genus level. (D) Rarefaction curves of HNC patients and controls. (E) Pan bacteria analysis HNC patients and controls. (F) Rarefaction curves of HNC patients and controls. (G) Pan bacteria analysis of HNCwP and HNCwoP. (H) Rarefaction curves of HNCwVS and HNCwoVS. (I) Pan bacteria analysis of HNCwVS and HNCwoVS. [file Image_1.tif]
